# Early central cardiovagal dysfunction after high fat diet in a murine model

**DOI:** 10.1038/s41598-023-32492-w

**Published:** 2023-04-21

**Authors:** Misty M. Strain, Liliana Espinoza, Stephanie Fedorchak, Erica L. Littlejohn, Mary Ann Andrade, Glenn M. Toney, Carie R. Boychuk

**Affiliations:** Department of Cellular and Integrative Physiology, Long School of Medicine, University of Texas Health San Antonio, 7703 Floyd Curl Drive, Mail Code 7746, San Antonio, TX 78229-3901 USA

**Keywords:** Hypertension, Neural circuits

## Abstract

High fat diet (HFD) promotes cardiovascular disease and blunted cardiac vagal regulation. Temporal onset of loss of cardiac vagal control and its underlying mechanism are presently unclear. We tested our hypothesis that reduced central vagal regulation occurs early after HFD and contributes to poor cardiac regulation using cardiovascular testing paired with pharmacology in mice, molecular biology, and a novel bi-transgenic mouse line. Results show HFD, compared to normal fat diet (NFD), significantly blunted cardio/pulmonary chemoreflex bradycardic responses after 15 days, extending as far as tested (> 30 days). HFD produced resting tachycardia by day 3, reflected significant loss of parasympathetic tone. No differences in bradycardic responses to graded electrical stimulation of the distal cut end of the cervical vagus indicated diet-induced differences in vagal activity were centrally mediated. In nucleus ambiguus (NA), surface expression of δ-subunit containing type A gamma-aminobutyric acid receptors (GABA_A_(δ)R) increased at day 15 of HFD. Novel mice lacking δ-subunit expression in vagal motor neurons (ChAT-δ^null^) failed to exhibit blunted reflex bradycardia or resting tachycardia after two weeks of HFD. Thus, reduced parasympathetic output contributes to early HFD-induced HR dysregulation, likely through increased GABA_A_(δ)Rs. Results underscore need for research on mechanisms of early onset increases in GABA_A_(δ)R expression and parasympathetic dysfunction after HFD.

## Introduction

Obesity not only carries a substantial health care burden^1^, it also increases the risk of cardiovascular diseases (CVD) such as arrhythmias, myocardial infarction, and heart failure^2^. Despite such clear connections between obesity and CVD, our understanding of the mechanism(s) responsible for poor cardiometabolic regulation remain limited in large part because it is not feasible to probe mechanistically in human models. Using animal models, excessive consumption of a high fat diet (HFD) is known to induce resting tachycardias^3–5^, which places individuals at risk for other CVDs, including heart failure^6–8^. Although the etiology of this resting tachycardia is not fully understood, animal model evidence implicates autonomic nervous system dysregulation as an underlying cause. The neurogenic component of HFD-induced cardiovascular dysfunction is thought to involve increased sympathetic drive, which rationalizes the use of clinical interventions targeting sympathetic hyperactivity^9–11^, despite reported negative outcomes such as poor symptom management and potentially serious side effects^12^.

This lack of clinically effective therapeutics highlights our incomplete understanding of the autonomic mechanism(s) responsible for parasympathetic (or vagal) regulation of cardiac function in disease. Several experimental observations indicate that long-term HFD decreases responsivity of cardiac vagal drive during homeostatic challenges^3,13–17^ and decreases in vagal components of heart rate (HR) spectral analysis^18^, providing compelling evidence that at least long-term consumption of HFD promotes poor cardiac vagal regulation. Despite our understanding that reductions in cardiac vagal tone are consistently linked to increased cardiovascular morbidity and mortality^19^, there is limited data on the early influence of HFD in facilitating poor vagal health, or perhaps more intriguingly poor vagal health as a major driver of negative consequences of HFD. This latter point made even more salient given the proposed role of vagal motor neurons to exercise tolerance^20^. Therefore, poor vagal control could facilitate further reductions in physical activity, thereby decreasing energy expenditure and leading to additional weight gain. Critical to generating a comprehensive neurogenic model of resting tachycardia and poor autonomic cardiac regulation is the emerging evidence that HFD robustly and rapidly modifies neural circuits^21,22^, suggesting that early central cardiac vagal dysregulation could promote the negative cardiac consequences of HFD.

Since cardio-vagal innervation restrains cardiac pacemaking rate by sino-atrial (SA) and atrio-ventricular (AV) nodal cells, reductions in vagal tonus promote not only resting tachycardia, but also a range of supraventricular tachy-arrhythmias, including atrial fibrillation. Loss of appropriate regulation of vagal motor output also disrupts critical homeostatic regulatory reflex processes, further contributing to CVD progression.

Cardiac vagal motor output is generated by brainstem cholinergic cardiac vagal motor neurons (CVNs) in the nucleus ambiguus (NA) that send axonal projections through the vagus nerve to a larger population of cardiac ganglion cells in the epicardial fat pad^23^. Despite their well-established cardioinhibitory influence through reductions in HR^24^, CVNs appear largely quiescent in vivo^25,26^ and in vitro^25^, indicating that cardiac vagal tonus does not result from large intrinsic pace-making discharges from CVNs, but rather from strong synaptic input and postsynaptic receptor sensitivity.

One type of postsynaptic receptors that mediates fast inhibitory neurotransmission is the type A gamma-aminobutyric acid receptors (GABA_A_R). GABA_A_Rs in CVNs determine resting HR^27^, and the magnitude of cardiorespiratory reflexes such as respiratory sinus arrhythmia^28^ and cardiopulmonary reflexes^29,30^. GABA_A_Rs are heteropentameric arrangements of varying subunit compositions. One subunit, the δ-subunit, confers persistent currents even when GABA concentrations are low through its high affinity and low desensitization kinetics^31,32^. Increased persistent currents have been identified in other models of metabolic dysregulation^33,34^. Therefore, GABA_A_Rs containing the δ-subunit (GABA_A_(δ)R) could be responsible for mediating reductions in vagal drive associated with HFD.

This study utilized chronic heart rate telemetry paired with pharmacological manipulation, autonomic reflex testing, and the development of a transgenic mouse model to test the hypothesis that decreased central vagal regulation contributes to cardiac chronotropic dysregulation during the first two weeks of HFD. It further hypothesizes that HFD-induced blunting of vagal HR control results from increased expression of GABA_A_(δ)R in vagal motor neurons.

## Methods

All experiments were performed on young adult male mice (25.9 ± 3.4 gm; 16.5 ± 8.1 weeks old). All strains were maintained on a C57/Bl6J background. Breeding C57/Bl6J mice were purchased from Jackson Research Laboratory and are now an established in-house colony at the University of Texas Health San Antonio (UTHSA). Animals were fed ad libitum and housed on a normal 14:10 light cycle in clear plastic cages (no more than 5 mice per cage) in a temperature-controlled (75 ± 3°F) room. All animal procedures were in accordance with NIH standards and ARRIVE guidelines for the care and use of laboratory animals and approved by the UTHSA Institutional Animal Care and Use Committee (IACUC).

### High fat feeding

Mice were randomly separated into two groups: high fat diet (HFD; 60 kcal% fat; D12492; Research Diet) or normal fat diet (NFD; 10 kcal% fat; D12450B). Food and water were provided ad libitum and diets were replaced every two days. Mice were maintained on these diets for the entire experimental timeline. Mice from the same litter were equally split between groups to control for litter effects. Weight and food intake were measured throughout experimental intervention. Diet lots were recorded for specific experiments, and diets were maintained in a cold room until used.

### Cardiovascular reflex testing

Mice were outfitted with a jugular catheter immediately before being tested for cardiovascular reflexes on either NFD or HFD after Day 3, 15 or Day 30. Mice were anesthetized with a cocktail of ketamine-xylazine (100 mg/kg:10 mg/kg). Surgical sites were shaved and aseptically prepared with betadine and alcohol. A small longitudinal incision was made about 1 cm from the sternum. The right external jugular vein was visualized with a surgical microscope and a 0.4–0.5 cm region of the vein exposed by blunt dissection, being careful to avoid damaging the vagus nerve. The jugular was gently lifted, and two small sutures placed underneath. Sutures were loosely tied around the cranial and caudal section of the vein. The cranial portion was tied to obstruct blood flow. A small incision was made into the vein using microscissors. The beveled end of a catheter (polyethylene tubing; PE10; Intramedic, 427401) pre-filled with saline was inserted into the jugular vein. Catheter was advanced 1 cm into the vein until the tip reached the atrium, and negative pressure applied to ensure the catheter was not obstructed. The caudal suture was then tied to secure catheter in place. The ventral incision was closed with suture (5–0). HR was monitored by telemetry devices (Data Sciences International, Inc.; model ETA-F10) placed in Lead II configuration and secured with 5–0 silk suture. Animals were recorded for a minimum of 15 min before reflex testing to allow the animal to stabilize after surgery. Once stable, animals were administered capsaicin through the jugular catheter, followed 5 min later by a saline injection to flush the catheter. Doses of capsaicin (10 μg/kg) were calculated based on weight and volumes taken from stock solution (5 ug/mL). In a subset of mice, animals were administered an intraperitoneal (i.p.) injection of atenolol (10 mg/kg) or scopolamine (1 mg/kg). For all drugs, at least a 15-min interval separated each dose. Animals with baseline HRs two standard deviations above or below mean HRs seen in control groups were excluded from analysis.

### Telemetry implants for HR recordings

Mice were implanted with HR telemetry devices to examine changes in HR during either HFD or NFD. Surgical sites were shaved and aseptically prepared with betadine and alcohol. Mice were anesthetized with isoflurane in oxygen (5% induction, then maintained at 2%). A vertical midline abdominal incision was made to open the intraperitoneal cavity. A telemetry device (ETA-F10; DSI) was inserted into the peritoneal incision. Using an 18-G syringe, the telemetry leads were tunneled so they protrude from the peritoneum. Leads were then placed in Lead II configuration and secured in place with 5–0 silk suture. The peritoneal cavity was sutured 5-0 silk suture and wound clips were used to close the abdominal incision and pain medication administered. Mice were then returned to their home cage and monitored postoperatively.

One week following telemetry implant and acclimation to HR telemetry recording station, animals were randomly assigned to either HFD or NFD. This was considered Day 0, and subsequent HR telemetry recordings were done on Day 3, 7, and 15. All recordings occurred between 12:00 and 18:00 h, and animals were singly housed during this time with water available ab libitum. In a subset of animals, the contribution of autonomic signaling to resting HR was examined using pharmacological manipulation on Day 15. For these experiments, animals were recorded for 1.5 h before being serially administered an intraperitoneal (i.p.) injection of atenolol (10 mg/kg) or scopolamine (1 mg/kg). Order of drug administration was randomized for each subject. Intrinsic HR was determined following the administration of both drugs.

### Vagal nerve stimulation

In a separate group of mice anesthetized with urethane (1.0 mg/kg) + alpha chloralose (0.1 mg/kg), a lead I ECG was recorded using needle electrodes inserted into each forepaw. The recorded ECG signal was directed to an AC amplifier equipped with a 60-Hz notch filter and half-amplitude filters set to a bandpass of 1–1000 Hz. The processed signal was digitized at a frequency of 1000 Hz. The right cervical vagus nerve was isolated from surrounding tissue, mounted on a bipolar stainless-steel wire (A-M Systems, Inc, 0.127-mm OD) electrode and insulated from body fluids with silicon elastomer (Kwik-Sil, WPI, Inc.). Ten minutes after administration of atenolol (10 mg/kg, i.v.), the distal cut end of the vagus nerve was stimulated with 0.2 ms square-wave pulses at frequencies ranging from 5 to 70 Hz. Stimulus intensity was set at an intensity that decreased HR by 10% of its baseline value when pulses were delivered for 5 s at the frequency of baseline HR (9–10 Hz).

### Generation of dual transgenic mice

To generate a colony of transgenic mice with a constitutive knock-out of the δ GABA_A_R subunit in cholinergic neurons (ChAT-δ^null^), floxed *Gabrd* (kind gift from Dr. Jamie Maguire) mice were crossed with a ChAT^IRES-cre^ mouse lines (B6.129S-*Chat*^*tm1(cre)Lowl*^/MwarJ; Jackson Research Labs). Primers and expected size for gene products associated with ChAT^IRES-cre^ transgene are listed in Table 1. Primers for genotyping of floxed *Gabrd* mice were as followed: 5′-GACTCCAGTTGCCAAGCCTTTAATTCC-3′ and 3′-CATCTGCCTGTACCTCCAATGCCTG-5′. Expected PCR product size was 543 bp for floxed *Gabrd* mice and 449 bp for wild-type mice.

**Table 1:** Primer sequences for ChAT^IRES-cre^.

Gene	Forward	Reverse	Size (bp)
IRES-Cre	TGGCTCTCCTCAAGCGTATT	AGGCAAATTTTGGTGTACGG	229
Chat-WT	CAATGGGTATGGAGCCTGTT	ACATGCCAGCTTCATGTGAG	397

### Immunohistochemistry

For immunohistochemical analysis, all mice were anesthetized with isoflurane until the toe-pinch reflex was absent. Mice were then perfused transcardially with ice-cold, heparinized phosphate-buffered saline (PBS; pH = 7.4) followed by ice-cold 4% paraformaldehyde. Brains were cryoprotected in 30% sucrose and sectioned at 40 µm in the coronal plane with a cryostat (Leica CM1860). Image acquisition parameters, including exposure time and illumination intensity, were identical across groups; brightness and contrast in all images were modified together and identically for figure presentation. Negative controls were run without primary antibody. All imaging was done with an Olympus BX43 microscope, and images were captured with a Retiga R6 CMOS digital camera (Teledyne Imaging, Tucson, AZ) using filters appropriate for the two fluorescent dyes.

To evaluate GABA_A_(δ)R expression, serial sections through the NA were rinsed with 1X PBS (pH 7.4) and placed at 4 °C under an incandescent light bulb for a minimum of 72 h to eliminate autofluorescence. Nonspecific immunoreactivity was blocked with 10% normal donkey serum (Jackson Immunoresearch, ref#017-000-121) in PBS. No detergent was used when noted to stain for GABA_A_(δ)R surface membrane expression. GABA_A_(δ)R expression was identified with primary rabbit anti-GABA_A_(δ)R (1:50, PhosphoSolutions; 868A-GDN) followed by secondary donkey anti-rabbit 488 Alexa Fluor (1:200, Invitrogen; A21206). To visualize ChAT + vagal motor neurons, a primary antibody against ChAT (goat anti-ChAT, 1:250; Sigma-Aldrich; AB144P) was used followed by secondary donkey anti-goat 568 Alexa Fluor (1:200, Invitrogen; A11057). Tissue from ChAT-δ^null^ and WT animals were run in parallel with the same antibody cocktails. In addition to negative controls stated above, robust labeling in the cerebellum served as a positive control due to consistently reported high δ-subunit expression in this brain region^35,36^.

For immunohistochemical analysis, the NA was identified with the use of a mouse brain stereotaxic atlas^37^, and ChAT + immunoreactivity was confirmed in this region. Expression of GABA_A_(δ)Rs was quantified in ImageJ (version 1.51j8). For each animal, 5–6 ChAT + neurons were selected from the NA based on their relatively large soma size and presence of a distinct nuclei (area devoid of ChAT + immunoreactivity). Each neuron was labeled as a region of interest (ROI) and mean intensity of the ROI was calculated for each neuron. Two different NA sections from each animal were quantified and the average intensity of all ROIs for each animal was used to quantify the total expression of the GABA_A_(δ)R subunit for each animal. Experimenter was blinded to treatment during analysis.

### Western blot analysis

Mice were anesthetized with isoflurane until the toe-pinch reflex was absent. The brains were excised and immediately placed in ice-cold artificial cerebral spinal fluid (aCSF; composition in mM: 124 NaCl, 3 KCl, 26 NaHCO3, 11 glucose, 1.3 CaCl2, and 1.3 MgCl2) bubbled with 95%O2-5%CO2. They were then sectioned at 300 µm in the coronal plane with a vibratome (Leica VT 1000S). Both right and left NA was isolated in a 1 mm-diameter punch by using a disposable tissue biopsy punch (Integra Miltex, 33-31AA). Samples from two mice were pooled together. Similar tissue collection procedures were performed in cerebellum to serve as a positive GABA_A_(δ)R control.

Samples were snap-frozen and stored at − 80 °C until homogenized in 100 µL of RIPA buffer (Sigma, ref#R0278) with 1% v/v each of protease inhibitor cocktail (Sigma, P8340), phosphatase inhibitor cocktail 2 (Sigma, P5726) and phosphatase cocktail 3 (Sigma, P0044). Protein concentration was confirmed with a BCA assay performed according to manufacturer instructions (Thermo Fisher, PI23227). Samples were then separated via SDS-PAGE and transferred to a nitrocellulose membrane via electroblotting. Presence of GABA_A_(δ)R was detected with rabbit anti-GABA_A_(δ)R (1:500; PhosphoSolutions) followed by secondary antibody goat anti-rabbit IRDye 800CW (1:5000; LI-COR, 926-32211). The housekeeping protein α-tubulin was used as a loading control and detected with primary rabbit anti-α-tubulin (1:1000; Cell Signaling Technology; 2144) followed by secondary goat anti-rabbit IRDye 800CW (1:5000; LI-COR, 926-32211). Blots were imaged with a blot reader (Odyssey CLx, LI-COR).

Following imaging, blot band density was measured with ImageJ (version 1.51j8). For each lane, a section containing no staining was selected and measured as background. An identically sized box was then drawn around each band at the protein size of interest (as indicated by the standardized protein ladder that was present), and band intensity was calculated as mean gray area. Each band was normalized to a loading control.

### Quantitative real-time polymerase chain reaction (qRT-PCR)

qRT-PCR was used to quantify and compare mRNA expression of GABA_A_R subunits (δ, α4, α5, and β3) across NA in mice fed NFD versus HFD for 15-days. Tissue containing NA was collected in the same manner as western blot samples, and immediately homogenized. RNA isolation was performed using the Qiagen kit and quantified by spectrometry measurement (Isogen; BioDrop µLite cat# 80-3006-50) of the A260/A280 ratio. An A260/A280 ratio in the range of 1.8 to 2.1 was considered pure. Samples exclusion criteria included a cycle threshold > 40 and an out of range A260/A280 ratio. No samples were excluded based on these criteria. qRT-PCR was performed by the Bioanalytics and Single-Cell Core (BASiC) at UT Health San Antonio using a Biomark HD system (Fluidigm). Fold change in mRNA expression was calculated using the formula for 2^−ΔΔCt^ with β-actin used as the house-keeping mRNA^38^. Although the 2^−ΔΔCt^ fold change is used in data presentation, statistical analysis of data was performed on ∆∆Ct values^39^.

### Data analysis

All HR recordings were analyzed offline using Spike2 software. For reflex responses, HR was averaged 30 s prior to capsaicin injection, and then again immediately following in a 10 s bin. HR reflex responses were normalized to pre-injection values. Resting HR was averaged from a stable 15 min recording two hours after the animals was placed in the recording room. HR responses to pharmacological testing were averaged for 5 min 15–20 min after drug injection. These were also normalized to the 5 min immediately preceding drug administration.

All data are presented as mean ± SEM. Graph creation and statistical analyses were performed with GraphPad Prism (version 9.1.2). Specific statistical analyses preformed are noted throughout in text. Briefly, Student’s t-tests were used when comparing two conditions. Paired and unpaired assessments were used when values were from the same subject or different subjects, respectively. To compare multiple conditions, when appropriate, a one-way (with or without repeated measures) ANOVA was used with post-hoc Tukey’s multiple comparisons or two-way (with or without) repeated measures ANOVA with post-hoc Sidak’s multiple comparisons. To examine the impact of HFD on vagal nerve stimulation by frequency, a repeated measure mixed-effects model ANOVA with post-hoc Sidak’s multiple comparisons was used. A Mann Whitney U test was used to compare two groups of non-parametric data. A linear regression was performed to determine if there was a relationship between body weight and specific cardiovascular parameters. Outliers were determined with a robust regression and outlier removal test with Q = 1%. Statistical significance was assigned a critical value of ≤ 0.05.

## Results

### HFD dampens capsaicin-mediated cardiopulmonary reflex

**Figure 1:**
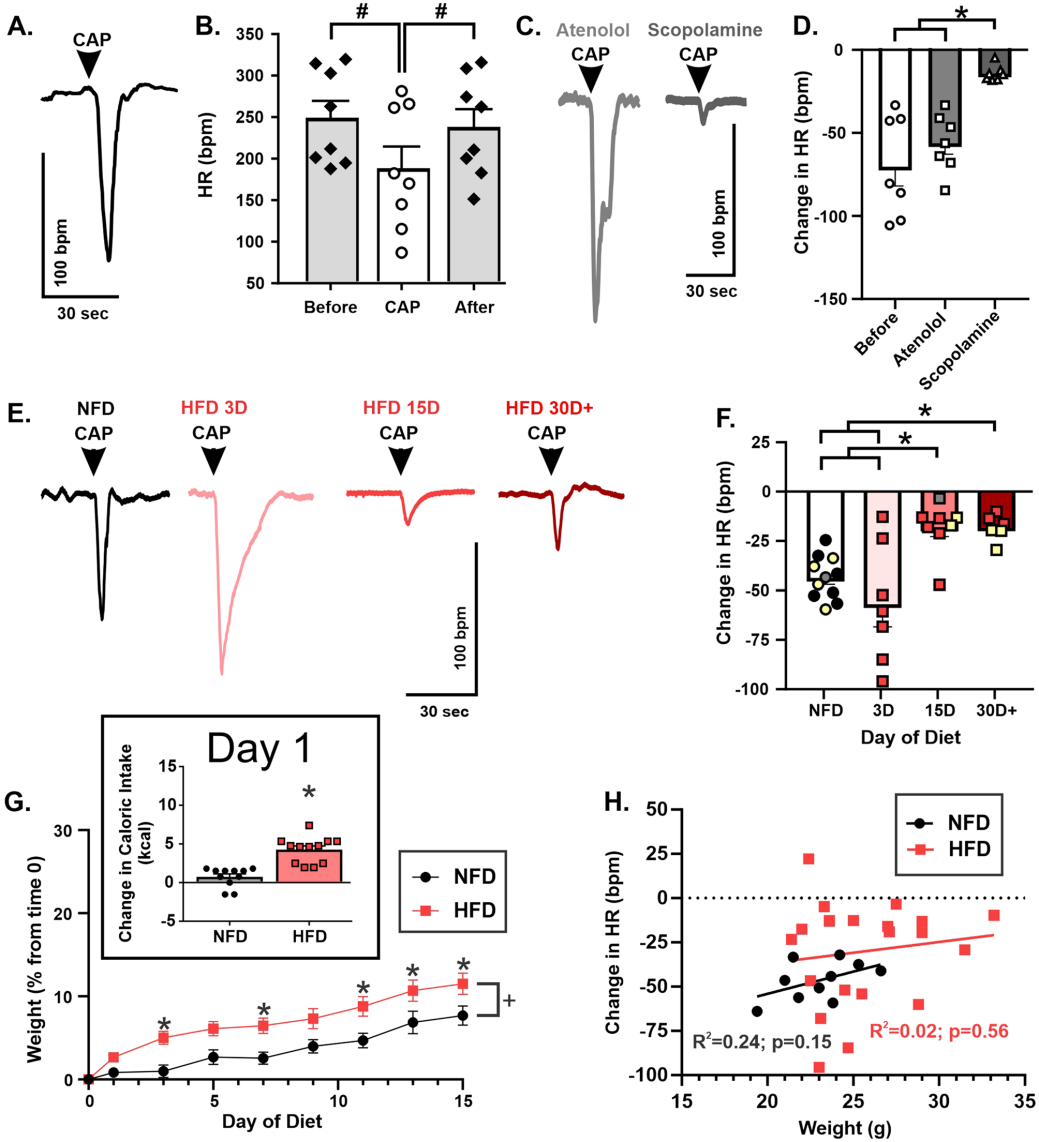
Capsaicin-mediated cardiopulmonary reflex is blunted by HFD. Representative HR responses to i.v. capsaicin (**A**). HR response (beats per minute; bpm) to capsaicin (10 μg/kg; n = 8 mice) (**B**). Representative HR responses after pre-treated with beta-adrenergic receptor antagonist, atenolol (10 mg/kg, i.p.) followed by muscarinic receptor antagonist, methylscopolamine (1 mg/kg; i.p.) (**C**). Mean HR responses to capsaicin were diminished after methylscopolamine, but not atenolol (n = 7) (**D**). Representative HR responses to capsaicin for each diet duration examined (**E**). Mice fed HFD for 15 days (n = 8) or more (n = 6) showed significantly reduced HR responses to i.v. capsaicin compared to NFD (n = 11) and Day 3 (n = 7); Overlaid symbols represent individual responses: yellow denotes animals under isoflurane, grey denotes urethane, all others are ketamine/xylazine (**F**). Percent change in weight from before start of diet (time 0) for the first two weeks for HFD (n = 11 mice) and NFD (n = 12 mice). Inserted graph illustrates the significant increase in food intake on Day 1 (no other time was significant, see results section for more details) (**G**). Weight on day of experiment did not predict HR responses in either NFD (n = 10 mice) or HFD (n = 20 mice) as determined by a simple linear regression (**H**). # denotes differences from before and after capsaicin (CAP); * denotes significant difference between diets indicated; + denotes significant difference from NFD regardless of day.

C57Bl/6 mice were tested for capsaicin-mediated cardiopulmonary reflex responses to jugular vein administration of capsaicin. As expected^40^, capsaicin elicited robust bradycardia, lowering HR from 249 ± 20 bpm before drug to 189 ± 26 bpm after treatment (n = 8; repeated measures one-way ANOVA with post-hoc Tukey’s post-hoc multiple comparisons; p = 0.0009; Fig. 1A-B). As expected, this response recovered quickly and therefore 40 s afterwards, HR (238 ± 21 bpm) was not significantly different from before treatment baseline (one-way repeated measures ANOVA with Tukey’s post-hoc multiple comparisons; p = 0.32; Fig. 1B).

To confirm that the elicited reflex bradycardia was vagally mediated, baseline bradycardic responses (i.e., no autonomic antagonists) were compared to those elicited after administration of either atenolol or atenolol + scopolamine in a subset of mice (n = 7; Fig. 1C). During baseline conditions, capsaicin elicited a 70 ± 12 bpm bradycardia, which was not significantly different from the capsaicin-induced bradycardia after atenolol (56 ± 7 bpm; one-way repeated measures ANOVA with Tukey’s post-hoc multiple comparisons; p = 0.15; Fig. 1D). However, scopolamine abolished capsaicin-induced bradycardia, indicating that the reduction in HR (14 ± 2 bpm; Fig. 1D) was significantly different from baseline (p = 0.009) and atenolol-treatment (p = 0.002). These data are in line with expected results from previous reports^40^, supporting that our intrajugular capsaicin-induced bradycardia was strongly mediated by increased vagal motor output.

To examine the effect of HFD on vagally-mediated cardiopulmonary reflex responses, again C57Bl/6 mice were randomly assigned to either NFD (n = 11) or HFD groups for 3 days (n = 7), 15 days (n = 8), or 30 + days (n = 6). Since we did not see a significant difference in HR responses to capsaicin with different anesthetics, animals given isoflurane, ketamine-xylazine, and urethane were combined for each group (denoted in Fig. 1F with different symbols). Mean bradycardic responses were not significantly different between NFD (43 ± 3 bpm) and Day 3 of HFD (57 ± 11; one-way ANOVA with Turkey’s post-hoc multiple comparisons, p = 0.39; Fig. 1E-F). However, by Day 15, mice fed HFD had a significantly blunted bradycardic response to capsaicin (18 ± 5 bpm) compared to NFD (p = 0.02) and Day 3 of HFD (p = 0.0008). This blunted response continued as long as tested, inclusive of 30 + days of HFD (18 ± 3 bpm) and remained different compared to NFD (p = 0.03) and Day 3 of HFD (p = 0.002). Additionally, the mean responses at Day 15 were not significantly different from mean responses at Day 30 + (p > 0.99), suggesting that while vagal reflex dysfunction occurs somewhere between Day 3 and 15 of HFD, no further functional change appears to occur thereafter.

During all investigations, mice were monitored for both body weight and food intake. As body weight and adiposity are both relevant factors in cardiovascular dysfunction, we chose to evaluate our data in the context of weight. HFD mice had a significant increase in food intake at Day 1 (4.3 ± 0.5 kcal; n = 12; Fig. 1G inset box) compared to their NFD counterparts at the same day (0.7 ± 0.4 kcal; n = 11; two-way repeated measures ANOVA with Sidak’s post hoc multiple comparisons; p < 0.0001). This increase in food intake at Day 1 was also significantly different from caloric intake in HFD mice at Day 0 (p < 0.0001). However, for the remaining duration of the two weeks examined, no differences in total calories consumed were observed between HFD and NFD. HFD also significantly increased weight gain compared to NFD (two-way repeated measure ANOVA with Sidak’s post hoc multiple comparisons; p = 0.008; Fig. 1G) with a significant interaction of day and diet (p = 0.005). Post-hoc analysis confirmed significant differences in the precent of weight gained between HFD and NFD at Day 3 (p = 0.02), 7 (p = 0.03), 11 (p = 0.02), 13 (p = 0.04) and 15 (p = 0.04). However, the maximum weight gain in any individual animal was 15.4% in the HFD. Therefore, during the time examined HFD mice gained more weight compared to NFD mice, but neither group would be considered “obese” (> 30%). To further confirm that HFD-induced blunting of the cardiopulmonary reflex bradycardic response was not related to body weight, the capsaicin-induced reduction in HR was plotted relative to body weight (Fig. 1H). Using a simple linear regression, there was no significant correlation between body weight and capsaicin-induced reflex bradycardia in HFD versus NFD mice (R^2^ = 0.24, p = 0.15). There was also no relationship between these variables in the HFD group (R^2^ = 0.02; p = 0.56). Therefore, weight did not predict the overall blunting of vagally-mediated reflex bradycardia.

### HFD causes resting tachycardia through altered autonomic signaling

**Figure 2:**
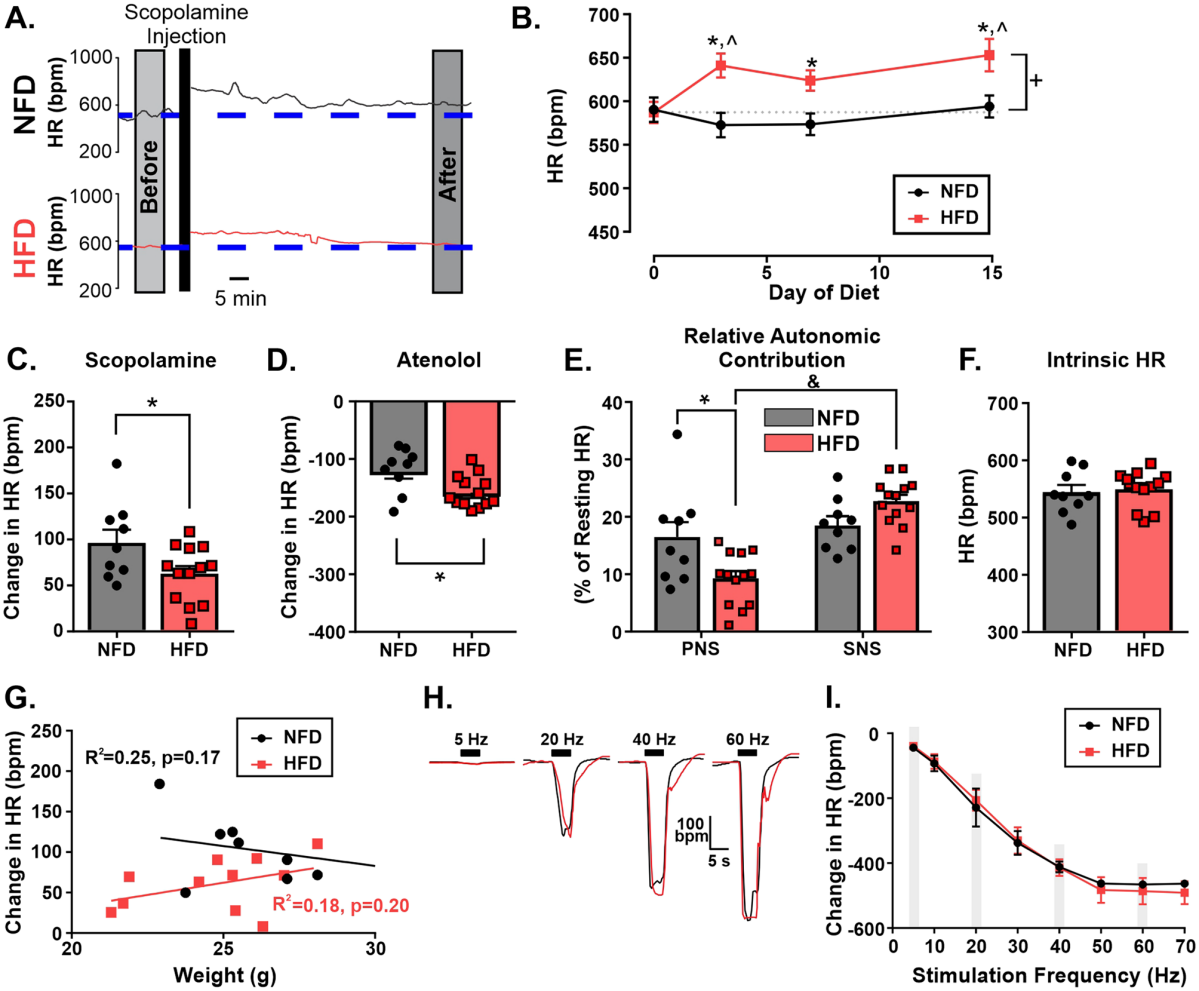
HFD induced a resting tachycardia and depressed vagal contribution to HR. These cardiovascular changes are centrally mediated. Representative HR trace from awake mice on Day 15 of diet before and after administration of methylscopolamine (**A**). Quiet resting HR was significantly increased after HFD (n = 15) compared to NFD (n = 13) (**B**). HFD (n = 13) reduced HR responses to methylscopolamine (**C**) and increased HR responses to atenolol (**D**) compared to NFD (n = 9). Change in HR after autonomic blockade as a percentage of initial resting HR change in HR demonstrated a significant reduction in parasympathetic tone, but not sympathetic tone (**E**). Intrinsic HR was not different between HFD (n = 13) and NFD (n = 9) (**F**). Linear regression showed no correlation between change HR or weight for either group (**G**). Representative image of HR change to nerve stimulation (**H**). Frequency stimulation curves in vagotomized mice did not differ with diet (NFD: n = 5; HFD: n = 5; p = 0.96). (**I**). * denotes significant difference from NFD; ^ denotes significant difference from time 0; + denotes significant difference from NFD regardless of day; & denotes significant difference from PNS within group.

We also examined the impact of HFD on resting HR over time focusing specifically on the first two weeks based on the results of reflex testing. Using a two-way repeated measures ANOVA, there was a significant interaction between diet x time (p = 0.01; Fig. 2A-B). Specifically, baseline HR did not differ between groups before starting diet (i.e., Day 0; Sidak’s post hoc multiple comparisons; p = 0.99; Fig. 2B). However, mice on HFD demonstrated a resting tachycardia at Day 3 (640 ± 54 bpm; n = 15), Day 7 (623 ± 46 bpm), and Day 15 (649 ± 78 bpm) compared to mice on NFD at Day 3 (571 ± 51 bpm; p = 0.003; n = 13), Day 7 (572 ± 45 bpm; p = 0.04), and Day 15 (596 ± 45 bpm; p = 0.01), respectively. While resting HR in NFD were not significantly different within group at any day examined, resting HR in mice fed a HFD at Day 3 and 15 was also significantly elevated from resting HR at Day 0 within group (Sidak’s post hoc multiple comparisons; p = 0.02 and p = 0.003, respectively).

To examine the role of the autonomic nervous system in HFD-induced tachycardia, changes in HR to i.p. administration of atenolol or scopolamine were determined. Mice on HFD demonstrated a significantly smaller increase in HR after scopolamine (63 ± 9 bpm; n = 13) compared to mice on NFD (98 ± 14 bpm; n = 9; unpaired Student t test, p = 0.04; Fig. 2C). However, mice on HFD also had a significantly larger reduction in HR after atenolol administration (-157 ± 8 bpm) compared to mice on NFD (-117 ± 11 bpm; unpaired Student t test, p = 0.01; Fig. 2D). To establish which change (decrease in parasympathetic or increase in sympathetic) might be a larger driver of the resting tachycardia, we compared the relative contribution of each branch by establishing the percent of baseline HR each branch contributed (change in HR after drug/HR before drugs). In mice on NFD, their relative contributions were approximately equal (scopolamine: 16.3 ± 0.3% compared to atenolol: 18.6 ± 0.01%; two-way repeated measure ANOVA with Sidak’s multiple comparison; p = 0.60; Fig. 2E). However, in mice on HFD, the contribution of vagal tone represented a significantly lower percent of resting HR (9.3 ± 0.01%) than atenolol (22.7 ± 0.01%; two-way repeated measure ANOVA with Sidak’s multiple comparison; p < 0.0001). As would be predicted by resting HR, vagal contribution to HR was also significantly lower in HFD mice compared to NFD (two-way repeated measure ANOVA with Sidak’s multiple comparison; p = 0.006). However, the atenolol-related contribution to resting HR was not significantly different between NFD and HFD (two-way repeated measure ANOVA with Sidak’s multiple comparison; p = 0.58). Importantly, intrinsic HR was not different between animals fed HFD (549 ± 9 bpm) or NFD (543 ± 12 bpm; unpaired Student t-test; p = 0.71; Fig. 2F). Finally, weight did not correlate with the change in HR after scopolamine for mice on NFD (R^2^ = 0.25, p = 0.17, simple linear regression) or mice on HFD (R^2^ = 0.18, p = 0.20; simple linear regression; Fig. 2G). Taken together, there is clear reorganization of autonomic control of HR at Day 15 of HFD feeding. As expected from the literature, sympathetic activity is higher in animals on HFD compared to NFD. However, these data provide evidence that vagal withdrawal is more important than sympathetic hyperactivity in the generation of resting HR at this early time point of HFD feeding.

### HFD did not inhibit the response of HR to graded electrical stimulation of the vagus efferent fibers

To assess the extent to which the observed reduction in vagal HR control reflects dysfunction in central circuitry relative to a reduction in the chronotropic response to efferent vagal activity, HR responses to graded electrical stimulation of right vagus nerve efferent fibers were recorded in mice where the vagus nerves had been bilaterally transected. Electrical stimulation of the distal segment of the transected vagus nerve elicited a frequency dependent bradycardia in all mice examined (repeated measure mixed-effects model ANOVA; stimulation frequency; p < 0.0001; Fig. 2H-I). Regardless of diet, increasing frequency of electrical stimulation of vagal motor fibers induced graded bradycardia (5 Hz: 41.7 ± 5.1 bpm; 10 Hz: 89.9 ± 15.6 bpm; 20 Hz: 217.8 ± 31.9 bpm; 30 Hz: 334.3 ± 25.7 bpm; 40 Hz: 412.9 ± 14.3 bpm; 50 Hz: 472.6 ± 19.7 bpm; 60 Hz: 477.2 ± 22.0 bpm and 70 Hz: 478.9 ± 19.5 bpm). There was, however, no significant interaction between diet and stimulation frequency (NFD: n = 5; HFD: n = 5; p = 0.98) and no difference in the bradycardic responses between diets regardless of the frequency of stimulation (p = 0.96). Therefore, stimulation of the vagal motor fibers to activate intracardiac ganglia and influence vagal drive at the SA node remained unaffected after two weeks of HFD.

### HFD increases GABA_A_(δ)R expression in NA

#### qRT-PCR for GABA_A_(δ)R expression

**Figure 3:**
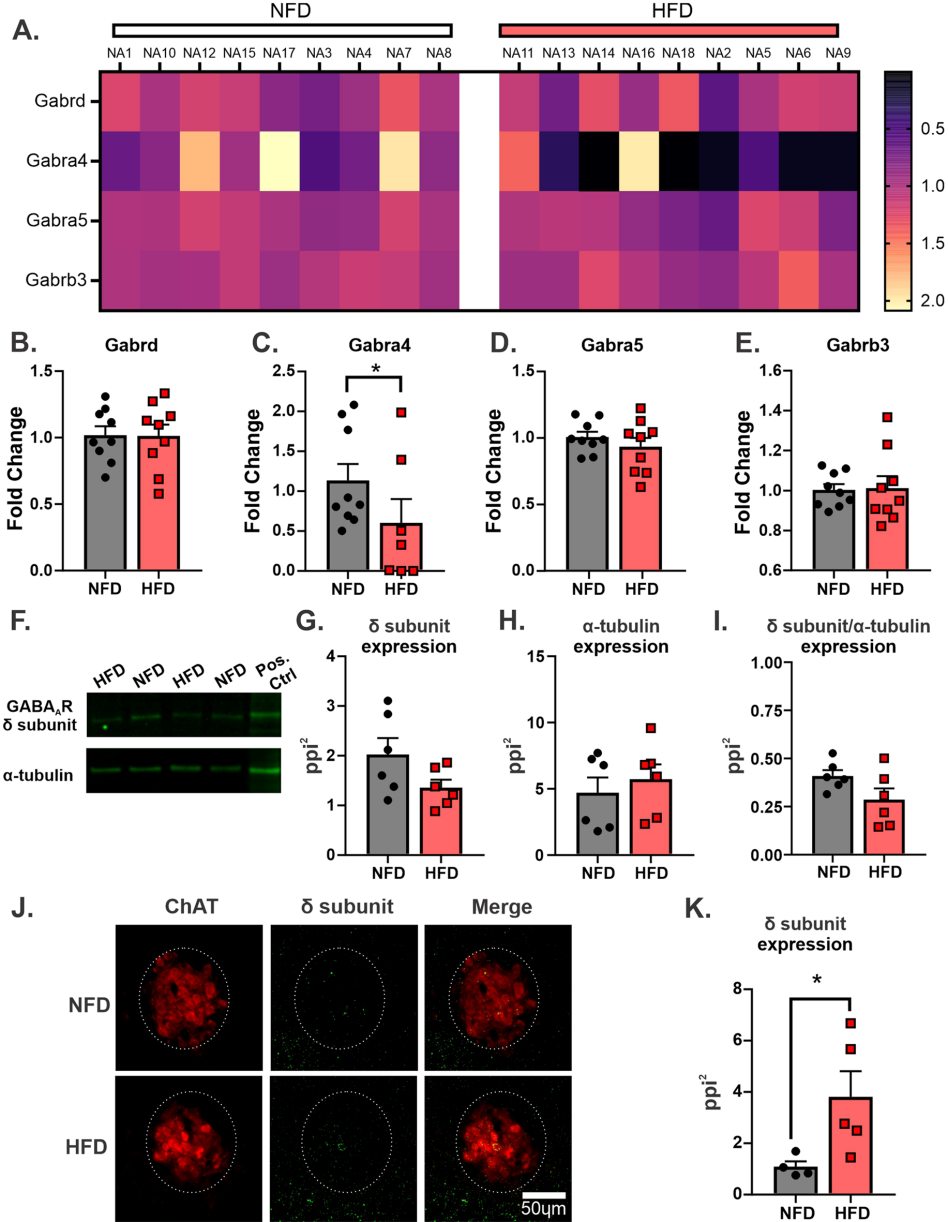
GABA_A_ (δ) receptor surface expression was increased after 15 days of HFD. Heat map of GABA_A_R subunits mRNA level in NA samples on Day 15 of diet (NFD: n = 9; HFD: n = 9) (**A**). Each column represents a single individual sample, both right and left NA are pooled. Mean mRNA levels of Gabrd (**B**), Gabra4 (**C**), Gabra5 (**D**), and Gabra3 (**E**) subunits. Gabra4 expression was significantly decrease after 15 Day of HFD compared to NFD. Representative western blot (**F**). No changes in GABA_A_(δ)R (**G**), α-tubulin loading control (**H**), and normalized GABA_A_(δ)R protein levels between HFD (n = 6 from 12 mice) and NFD (n = 6 from 12 mice) on Day 15 (**I**). Original western blot is presented in Supplementary Fig. 1. Representative immunohistochemical images co-strained for ChAT (red) and GABA_A_(δ)R (green) in NA on Day 15 of diet (**J**). GABA_A_(δ)R surface expression in ChAT + neurons within NA was increased after HFD (n = 5) compared to NFD (n = 4) (**K**). * denotes significant difference between diet.

qRT-PCR was used to determine whether increases in GABA_A_ receptor expression could reflect the increased inhibition of vagal motor output. Since a previous report using models of metabolic dysfunction suggested that GABA_A_ receptors containing the δ-subunit (GABA_A_(δ)R) were increased^33^, changes in the δ-subunit and its traditional pairing partners the α4-, α5-, and β3-subunit were examined (Fig. 3A). There was no significant difference in mRNA expression between NFD (n = 9) and HFD (n = 9) for the δ-subunit (1.0 ± 0.1 fold change in NFD versus 1.0 ± 0.1 fold change in HFD; Student’s unpaired t-test, p = 0.9592; Fig. 3B). The α4 subunit, however, did have significantly lower expression in animals fed a HFD (0.6 ± 0.3 fold change) compared to NFD (1.1 ± 0.2 fold change; Student’s unpaired t-test, p = 0.03; Fig. 3C). There was also no significant difference for the α5 subunit (1.0 ± 0.04 fold change in NFD versus 0.9 ± 0.06 fold change in HFD; Student’s unpaired t-test, p = 0.3652; Fig. 3D) or the β3 subunit (1.0 ± 0.03 fold change in NFD versus 1.0 ± 0.06 fold change in HFD; Student’s unpaired t-test, p = 0.8977; Fig. 3E). Since these values are normalized to β-actin, we also evaluated whether average cycle threshold of β-actin was comparable between NFD and HFD. There was no significant difference between β-actin cycle threshold from the HFD group (6.5 ± 0.3 cycle threshold) compared to NFD (6.0 ± 0.1 cycle threshold; Student’s unpaired t-test, p = 0.0972).

#### Western Blot for GABA_A_(δ)R expression

Since mRNA expression of the GABA_A_(δ)R was not significantly different, we used western blot analysis to examine whether HFD increased the overall protein expression of GABA_A_(δ)R in NA between NFD animals (n = 6 individual samples from a total of 12 mice; two mice/sample) and HFD animals (n = 6 individual samples from a total of 12 mice; two mice/sample; Fig. 3F). Similar to transcriptional expression, there was no significant difference in GABA_A_(δ)R expression between animals consuming NFD (2.024 ± 0.3 pixel intensity per square inch [ppi^2^]) or HFD (1.359 ± 0.2 ppi^2^; Student’s unpaired t-test, p = 0.1000; Fig. 3G). The loading control, α-tubulin, expression was not different between NFD (4.711 ± 1.1 ppi^2^) and HFD (5.734 ± 1.1 ppi^2^; Student’s unpaired t-test, p = 0.5360; Fig. 3H). Finally, expression in each animal was also normalized to α-tubulin expression as a loading control. Again, there was no significant difference in the ratio of GABA_A_(δ)R to α-tubulin between NFD (0.4089 ± 0.1 ppi^2^ ratio GABA_A_(δ)R/α-tubulin) and HFD groups (0.2864 ± 0.1 ppi^2^; Student’s unpaired t-test, p = 0.4659; Fig. 3I). Original gels are presented in Supplementary Fig. 1.

#### Immunohistochemistry for GABA_A_(δ)R expression

To determine if changes in GABA_A_(δ)R expression were related to surface expression, immunohistochemical analysis was examined (Fig. 3J). This protocol specifically omitted any detergent from washes to ensure that antibody did not penetrate the cellular membrane. Tissue was also co-stained for ChAT to identify motor neurons in the NA. Therefore, GABA_A_(δ)R subunit expression in ChAT + neurons in the NA was examined. Quantification of fluorescent intensity of GABA_A_(δ)R determined a significant increase in HFD animals (3.811 ± 1.0 ppi^2^; n = 5) compared to NFD animals (1.088 ± 0.2 ppi^2^; n = 4; unpaired Student’s t-test, p ≤ 0.05; Fig. 3K). Taken together, HFD for two weeks increases the surface expression of GABA_A_(δ)R in the NA, but not likely through transcriptional or translational upregulation.

#### Cardiovascular effects of HFD are attenuated in ChAT-δ^null^ mice

**Figure 4:**
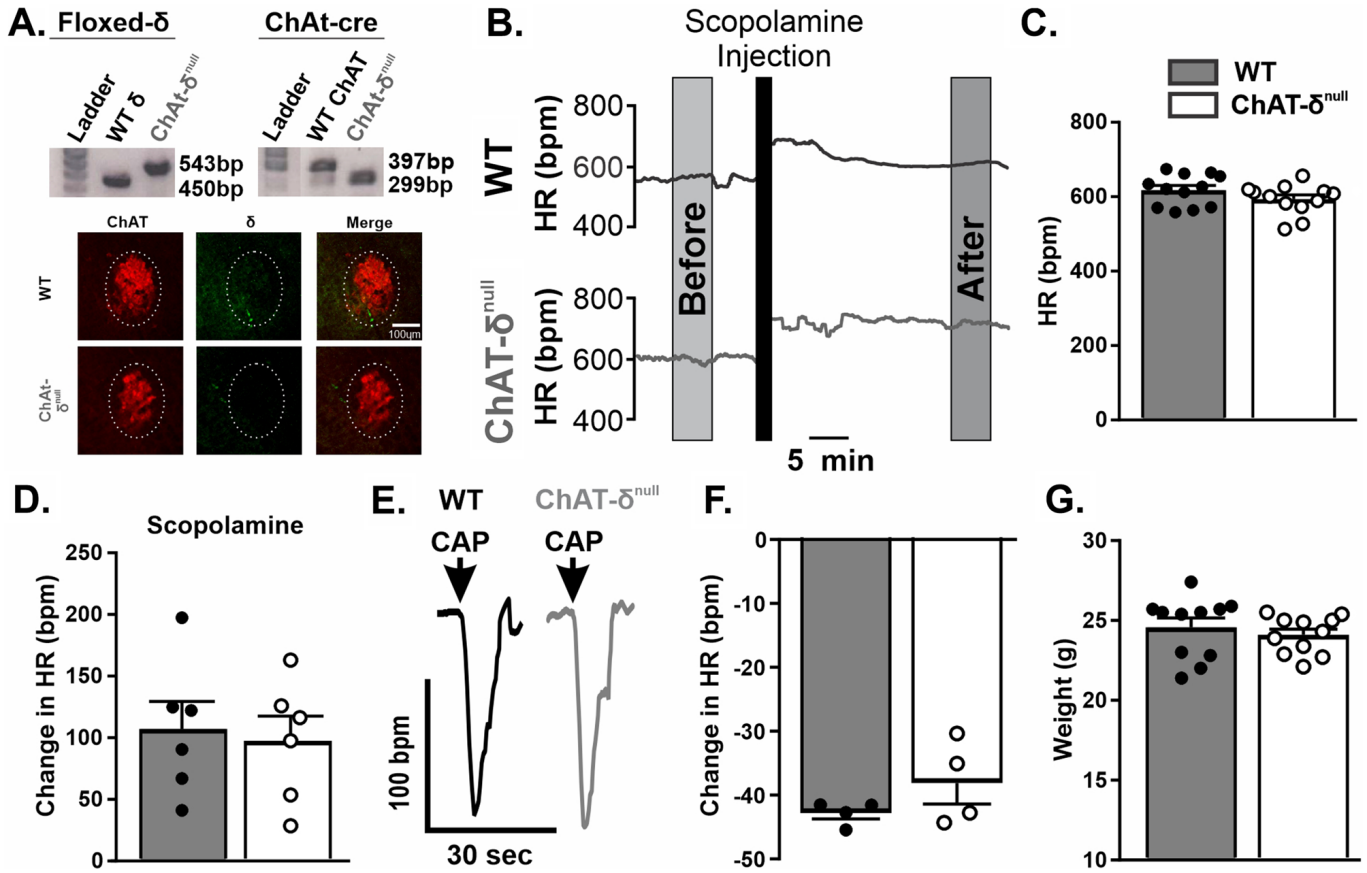
Novel ChAT-δ^null^ mice strain showed no differences in resting HR and reflex responses compares to C57/BL6 wild-types (WTs). ChAT-δ^null^ transgenic mice were generated by cross breeding floxed Gabrd and ChAT^cre^ mouse lines. Genotyping with in-house PCR confirmed that mice classified as ChAT-δ^null^ were homozygous for both gene cassettes. Qualitative immunohistochemical imaging confirmed that ChAT-δ^null^ had reduced GABA_A_(δ)R protein expression compared to WT mice in the NA (**A**). Original PCR gel is presented in Supplementary Fig. 2. Knock down of GABA_A_(δ)R was confirmed using PCR and immunohistochemistry (**A**). Representative traces (**B**) and mean HR (**C**) in awake WTs (n = 12) and ChAT-δ^null^ mice (n = 12) showed no differences between groups. Change in HR in response to methylscopolamine was not different between strains (WT: n = 6; ChAT-δ^null^: n = 6) (**D**). Representative traces (**E**) and mean HR responses (**F**) to capsaicin in WT (n = 4) and ChAT-δ^null^ mice (n = 4) show no differences between groups. Both mouse strains show similar weight on NFD (WT: n = 12; ChAT-δ^null^: n = 12) (**G**).

Genotyping with in-house PCR was able to discriminate between wild-type (WT) and all transgenic mice (Fig. 4A). Therefore, genotyping confirmed that mice classified as ChAT-δ^null^ were homozygous for both gene cassettes (Fig. 4A). Qualitative immunohistochemical imaging confirmed that ChAT-δ^null^ had reduced GABA_A_(δ)R expression compared to WT mice in the NA (Fig. 4A). To determine if ChAT-δ^null^ mice exhibited any overt cardiovagal phenotypes normally, these mice were compared to WT C57Bl/6. At eight weeks of age, there were no differences in resting HR in ChAT-δ^null^ (594 ± 12 bpm; n = 12) compared to WT mice (619 ± 14 bpm; n = 12; unpaired Student t test; p = 0.17; Fig. 4B-C). In a small subset of animals, scopolamine was administrated. There was no significant difference in HR responses to scopolamine between ChAT-δ^null^ (98 ± 20 bpm; n = 6) and WT mice (107 ± 22 bpm; n = 6; unpaired Student t-test; p = 0.76; Fig. 4B and 4D). There was also no significant difference in intrinsic HR in ChAT-δ^null^ (535 ± 10 bpm; n = 6) and WT mice (542 ± 17 bpm; n = 6; unpaired Student t-test; p = 0.70). In ChAT-δ^null^ mice, jugular vein administration of capsaicin elicited a bradycardia (38 ± 3 bpm; n = 4) that was not significantly different from WT bradycardias (43 ± 1 bpm; n = 4; unpaired Student t test; p = 0.22; Fig. 4E-F). Finally, there was no significant difference in weight (24.1 ± 0.4 g in n = 12 ChAT-δ^null^ mice vs. 24.6 ± 0.6 g in n = 12 WT mice; unpaired Student t test; p = 0.50; Fig. 4G).

**Figure 5:**
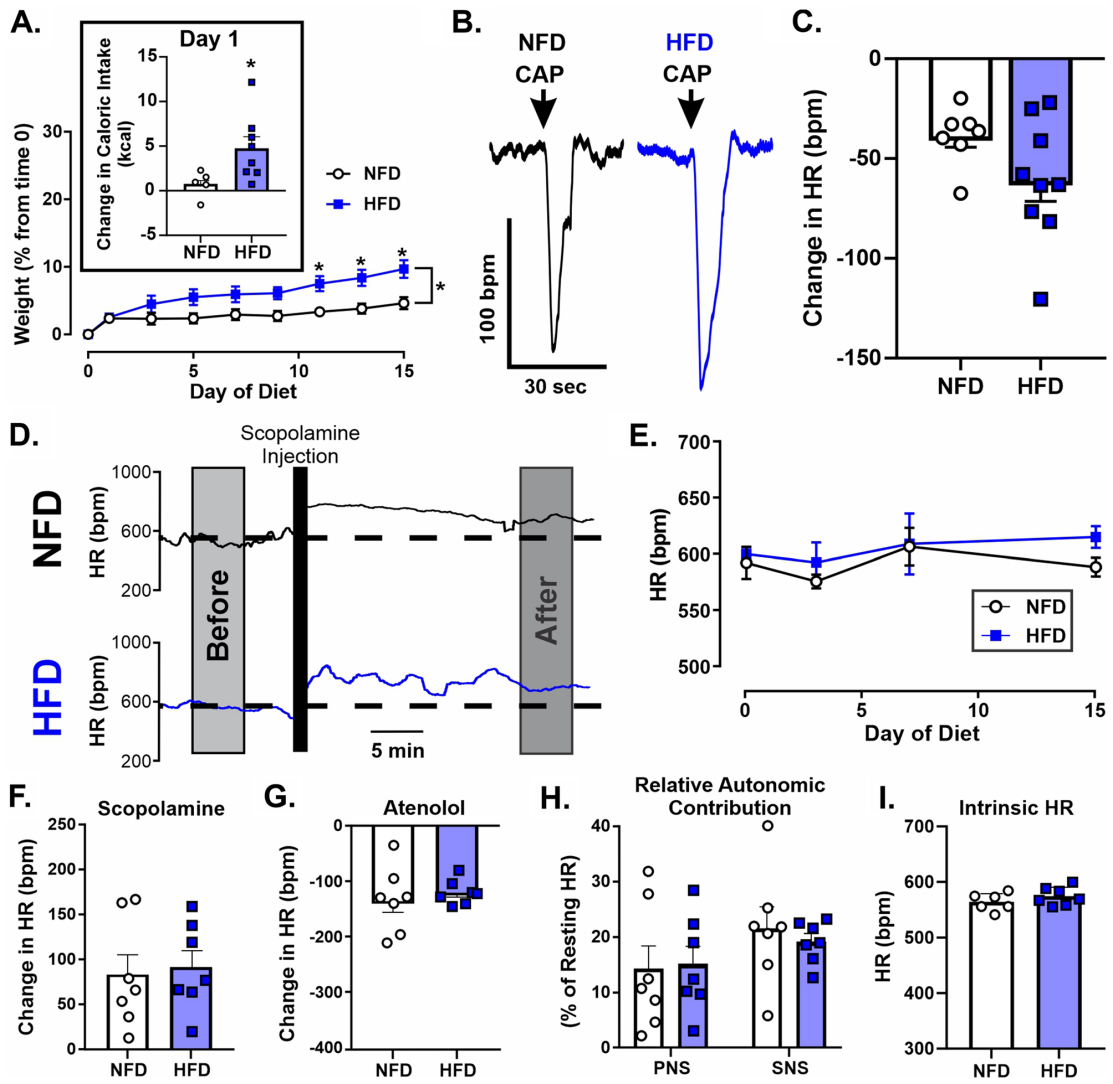
ChAT-δ^null^ mice were not significantly different then NFD controls after 15 days of HFD. HFD increased percent body weight over time and Day 1 food intake in ChAT-δ^null^ mice (n = 8) compared to NFD (n = 7) (**A**). Representative HR trace of capsaicin-induced cardiopulmonary chemoreflex response on Day 15 of either NFD or HFD (**B**). Mean HR change to cardiopulmonary chemoreflex was not significantly different between HFD (n = 9) and NFD (n = 7) in ChAT-δ^null^ mice (**C**). Representative HR trace from awake ChAT-δ^null^ mice on day 15 (D). Quiet resting HR over time (**E**), and response to methylscopolamine (**F**) and atenolol (**G**), were not different between ChAT-δ^null^ mice on HFD (n = 7) and NFD (n = 7). Autonomic contribution to resting HR (**H**) and intrinsic HR (**I**) were also not different in ChAT-δ^null^ mice on HFD (**E**) compared to ChAT-δ^null^ mice on NFD. * denotes significant difference between diet.

Having confirmed a lack of robust physiological phenotypes compared to WT mice under resting conditions, we further quantified changes after HFD challenge. ChAT-δ^null^ mice on HFD weighed significantly more than ChAT-δ^null^ mice on NFD (n = 7–8 for each group; repeated measure two-way ANOVA; diet: p = 0.01; Fig. 5A). Weight gain in ChAT-δ^null^ mice on HFD (10 ± 2%) was similar to weight gain in WT mice fed a HFD (7 ± 3%; unpaired Student t-test, p = 0.45). There was also a significant interaction of diet by day (p < 0.0001), and post hoc analysis (Sidak’s multiple comparisons) determined that increase in weight was significant specifically at days 11, 13, and 15. Comparing Day 1 caloric intake, ChAT-δ^null^ mice ate significantly more kilocalories (n = 7–8 for each group; unpaired Student t test; p = 0.04; Fig. 5A). We confirmed that ChAT-δ^null^ on HFD had similar cardiopulmonary reflex-mediated bradycardias (61 ± 10 bpm; n = 9) compared to their counterparts on NFD (39 ± 6 bpm; n = 7; unpaired Students t-test, p = 0.10, Fig. 5B-C). Additionally, ChAT-δ^null^ failed to demonstrate HFD-induced resting tachycardia at any time point examined (NFD: n = 7; HFD: n = 7; repeated measure two-way ANOVA with Sidak post hoc multiple comparisons; p = 0.83; Fig. 5D-E). As expected, since there was no change in resting HR, changes in HR after i.p. administration of scopolamine were not statistically different in HFD (92 ± 18 bpm; n = 7) compared to NFD (82 ± 23 bpm; n = 7; unpaired Students t-test, p = 0.80; Fig. 5F). Mice on HFD also did not significantly change their HR response to atenolol administration (119 ± 8 bpm; n = 7) compared to mice on NFD (132 ± 23 bpm; n = 7; unpaired Students t-test, p = 0.60; Fig. 5G). An examination of the relative contribution of each branch of the autonomic nervous system (PNS and SNS) revealed no significant difference between HFD or NFD in either PNS or SNS (diet X autonomic branch interaction: p = 0.42; repeated measure two-way ANOVA; Fig. 5H). Finally, intrinsic HR was not different between animals fed HFD (571 ± 6 bpm; n = 7) or NFD (561 ± 6 bpm; n = 7; unpaired Student t-test; p = 0.27; Fig. 5I). Taken together, genetic deletion of GABA_A_(δ)Rs from ChAT + neurons ameliorated the blunting of vagal reflex responses, increase in resting HR, and decreased vagal tone that accompanied HFD feeding in mice.

## Discussion

The present study examined the impact of HFD on vagal control of HR using a number of different approaches, including pharmacological manipulations, molecular biology and a novel transgenic mouse model. Initial investigations found that HFD blunted the HR response to vagal reflex activation as early as Day 15 and this continued for as long as examined (Day 30 +). This blunting of reflex vagal bradycardia was associated with resting tachycardia as well as low vagal tone, as determined through pharmacological approaches. Importantly, cardiac ganglia and nodal cell function appeared normal given that HR responses to electrical stimulation of motor axons of the right vagus nerve were not different between NFD and HFD. Increased GABAergic inhibition from increased membrane expression of GABA_A_(δ)R in the NA, a major cardioinhibitory brain region, likely contributed to the observed reduction in vagal drive. Increased inhibition does not appear to result from any detectable changes in the apparent rate of transcription or translation of the GABA_A_(δ)R in the NA. Finally, we determined that although ChAT-δ^null^ mice on HFD gained weight similar to WT mice, they were resistant to the impact of HFD on examined cardiac parameters, consistent with GABA_A_(δ)R playing a key role in negative effects of HFD on cardiac health. Taken together, these data support the hypothesis that HFD reduces central vagal motor drive, at least in part, through increased GABA_A_(δ)R functional expression in NA.

While we are only beginning to elucidate the complexity of sensory afferent signaling through newly developed advances in genetic analysis of vagal afferent neurons^41,42^, it has long been known that activation of vagal afferents expressing transient receptors potential vanilloid type 1 (TRPV1) channels induces a cardiopulmonary chemoreflex since intrajugular capsaicin (a TRPV1 agonist) stimulates apnea generation, vagally-mediated bradycardia and a depressor response^40,43^. Therefore, as expected, administration of capsaicin here induced a robust bradycardia in mice that required intact muscarinic acetylcholine receptor signaling. We are, however, the first to determine that HFD for two weeks significantly blunts capsaicin-induced bradycardias consistent with vagal motor output impairment. Although this blunted vagal response was not present 3 days after HFD, it was present by Day 15 and persisted as far out as tested (30 + days).

While we are the first to demonstrate blunted reflex bradycardia by short-term HFD, there are some inconsistencies with previous research regarding HFD-induced resting tachycardia. Indeed, some studies reported no change in HR^44,45^ while others report a resting tachycardia similar to that seen here^5,46–48^. Notably, these latter studies all recorded HR using methods that require limited experimenter interaction with test subjects and minimize animal stress, such as radiotelemetry, and therefore, our results are consistent with most previous reports, and emphasize the utility of telemetry recordings when examining HFD’s impact on cardiovascular regulation. It is also established that HFD-induced resting tachycardia is most prominent during the sleep phase^49^. The present results were recorded during day light hours (a mouse’s prominent sleep phase), providing further similarities to previous literature that HFD-induces a significant resting tachycardia even after sub-chronic feeding (> 15 days).

It is interesting to consider data on resting tachycardia and blunted reflex bradycardia together. Since HFD induces a resting tachycardia at Day 3 (relative to NFD), with no significant change in reflex bradycardia (or perhaps even an increase), we can speculate that vagal motor neurons supporting resting HR and those that participate in reflex control of HR mediated by TRPV1 + afferents form distinct, non-overlapping circuits. Moreover, those vagal motor neurons supporting resting HR may be uniquely susceptible to modulation by early consumption of HFD or lack compensatory mechanism(s). Specifically, increased glutamatergic synaptic neurotransmission is seen between 3–5 days of HFD in vagal motor neurons of the dorsal motor nucleus of the vagus^21^. Since cardiac vagal reflexes are dependent on glutamatergic drive from the NTS, a large excitatory drive could be either a compensatory mechanism for greater inhibition of cardiovagal motor neurons or a separate process. However, since the blunted vagal reflex (and resting tachycardia) is the persisting phenotype, the increase in excitatory drive appears to eventually fail (or fails to compensate) by Day 15, and apparently does not return with continued consumption of HFD. This failure of excitatory drive to compensate for increased GABA inhibition would also explain dampening of vagal motor neuron firing frequency after 3 months of HFD feeding^50^.

Several critical questions remain. Namely how specific or generalizable is the HFD-dysregulation to certain microcircuits. It has long been theorized that most second order NTS neurons represent a specific circuit since most NTS neurons receive inputs from only one or two afferent fibers^51,52^. However, whether a given NTS neuron communicates only with a single cardiac vagal motor neuron is currently unknown, and emerging evidence has only recently provided insight enabling construction of “projection models” of vagal motor neurons themselves^53,54^. Therefore, future work should investigate a wider range of vagally-mediated reflexes, and critically as technology advances, aim to construct the basic circuit building blocks of such critical homeostatic regulating networks. For example, it remains possible that incomplete baroreflex resetting, and subsequent decreases in glutamatergic drive to cardiac vagal motor neurons contributes to resting tachycardia. Despite existing questions regarding the structure of reflex vagal circuitry, our data clearly indicate that HFD-induced vagal hypoactivity is an early predictor of poor cardiac autonomic regulation.

Regardless of whether these are separate circuits with unique susceptibilities to modulation by HFD, our transgenic ChAT-δ^null^ mouse line was resistant to the impact of HFD on both resting HR and vagal reflex responses. Therefore, while the divergent timeline suggests that regulation of resting HR and of capsaicin-induced reflex HR response could arise from different central circuits, the surface expression of GABA_A_(δ)R must be critical for the impact of HFD on cardiac vagal regulation. Our results are similar to others and suggest that GABA_A_(δ)R expression is upregulated during metabolic challenges in vagal motor neurons^33^, and that neuronal membrane expression (and not overt transcription) are likely the mechanism of such increased functional activity^33^. Our results also extend earlier findings to include impacts of HFD on vagal motor neurons in the NA. Since GABA_A_(δ)Rs generally are subject to few covalent modulatory signals impacting their gating^55^, we suggest that HFD and perhaps chronic disease states instead modulate their expression and/membrane localization.

Unfortunately, little work investigating the regulation of GABA_A_(δ)Rs has been conducted in brainstem nuclei, and this should be a focal point for future work. Increases in the activity of protein kinases, specifically PKC and PKA, are implicated in altered membrane expression of GABA_A_(δ)R^55^. GABA_A_(δ)Rs undergo constitutive recycling from the cell membrane. Although not *required* for entrance into the membrane, protein kinase-dependent phosphorylation of GABA_A_(δ)Rs allow longer dwell times within the membrane by limiting their removal^56^. This relationship between GABA_A_(δ)R and protein kinases is likely true in vagal motor neurons as well since protein kinase-dependent phosphorylation of receptors containing the δ-subunit is low under normal conditions, but activation of PKC robustly increases GABA_A_(δ)R functional activity^57^. Since only surface expression and not mRNA or whole protein content were altered in the present study, our results are consistent with HFD impacting vagal motor neurons in NA through increases in serine/threonine phosphorylation of GABA_A_(δ)R. Since GABA_A_(δ)Rs themselves do not contain strong phosphorylation sites^56^, post-translational regulation of GABA_A_(δ)Rs to increase their localization to the neuronal membrane is theorized to rely on alternative subunits, namely α4 and/or β3^58^. Although increased GABA_A_(δ)Rs at the membrane provide a mechanistic link to HFD-induced deficits in vagal output, it is also possible that decreased α4 level in NA, as seen in the present study, may be a contributing upstream mechanism. Since the α4-subunit is implicated in the appropriate assembly of GABA_A_(δ)Rs, a decrease in the α4-subunit by HFD could cause GABA_A_(δ)Rs to “misassemble” with alternative subunit partners, resulting in increased membrane expression. However, α4-subunit phosphorylation by PKC is implicated in the maintenance of GABA_A_(δ)Rs in the membrane^59^, and reduced assembly of α4 within GABA_A_(δ)Rs would predict higher constitutive recycling and smaller tonic currents. Therefore, it remains possible that decreased α4-subunit levels are a compensatory mechanism since experiments using various GABA_A_R-subunit-deficient mice indicate that modifying transcript levels of preferential subunit binding partners does influence transcript levels of other subunits^60–62^. Understanding the stoichiometry of GABA_A_R subunit assembly will be critical to our understanding of the impact of HFD on vagal motor neurons, and brain function more broadly.

Regardless of mechanism, increasing GABA_A_(δ)R persistent inhibitory current can dramatically alter neuronal information processing. Because reflex responses critically depend on precise timing of synaptic inputs, the long duration of inhibition conferred by GABA_A_(δ)R would persistent well beyond any transient reflex-activated GABAergic input. A second GABAergic event could then encode greater inhibition than would normally be expected. Since GABA_A_(δ)Rs are overexpressed at the membrane, this inappropriate signaling would persist over long time scales, and vagal output would be unresponsive to changes in synaptic input needed for rapid vagal regulation of HR.

The present study was conducted in only male mice since there are well established sex differences in fat consumption^63,64^ and fat metabolism^3,65–67^. With the negative consequences of HFD on vagal function defined in male mice, sex differences can be probed to determine key mechanistic insights into the establishment of the consequences of HFD. Therefore, future studies will examine whether female mice show a similar timeline for vagal dysfunction after HFD. In particular, previous work from our laboratory suggests that vagal motor neurons of the dorsal motor nucleus of the vagus increase GABAergic tonic currents (putatively δ-subunit containing) during diestrus through the progesterone-derivative, allopregnanolone^57^. HFD also increases the amount of systematic progesterone in females at 4 weeks^68^, likely contributing to prolonged diestrus^69^. Therefore, female mice on HFD may not show consistent cardiovascular dysfunction until diet prolongs diestrus leading to prolonged periods of large δ-subunit-mediated GABAergic tonic currents. However, investigations into sex differences in NA motor neurons are limited and additional future experiments should examine this as a mechanism of HFD and sex differences in vagal regulation.

In summary, this study revealed that HFD impairs cardiac vagal output both at rest and during cardiovascular reflex induction. This lowered vagal activity is more prominent than increased sympathetic activity; and importantly is centrally mediated. We speculate that the inhibition of cardiac vagal motor output occurs through increased membrane expression of the GABA_A_(δ)R, and that correcting this could abolish the ability of HFD to decrease vagal motor activity after HFD consumption. Future studies will be needed to more completely examine intracellular mechanism(s) behind the upregulation of GABA_A_(δ)Rs in vagal motor neurons in the pathogenesis of metabolism-related cardiovascular disease.

## Supplementary Information

Supplementary Information 1.

Supplementary Information 2.

Supplementary Information 3.

## Data Availability

The datasets generated during and/or analyzed during the current study are available from the corresponding author on reasonable request.
