# Supplementary figures and images for: Early central cardiovagal dysfunction after high fat diet in a murine model
Source: Sci Rep. 2023 Apr 21;13:6550. doi: 10.1038/s41598-023-32492-w (PMC10121716; doi:10.1038/s41598-023-32492-w)

# Delta subunit

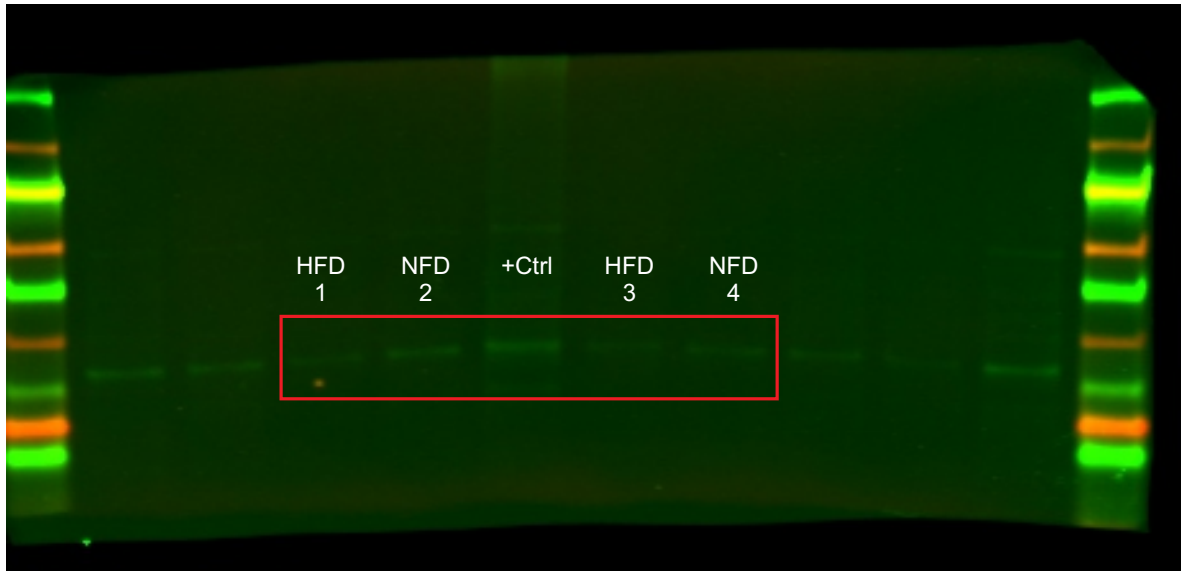

# Alpha tubulin

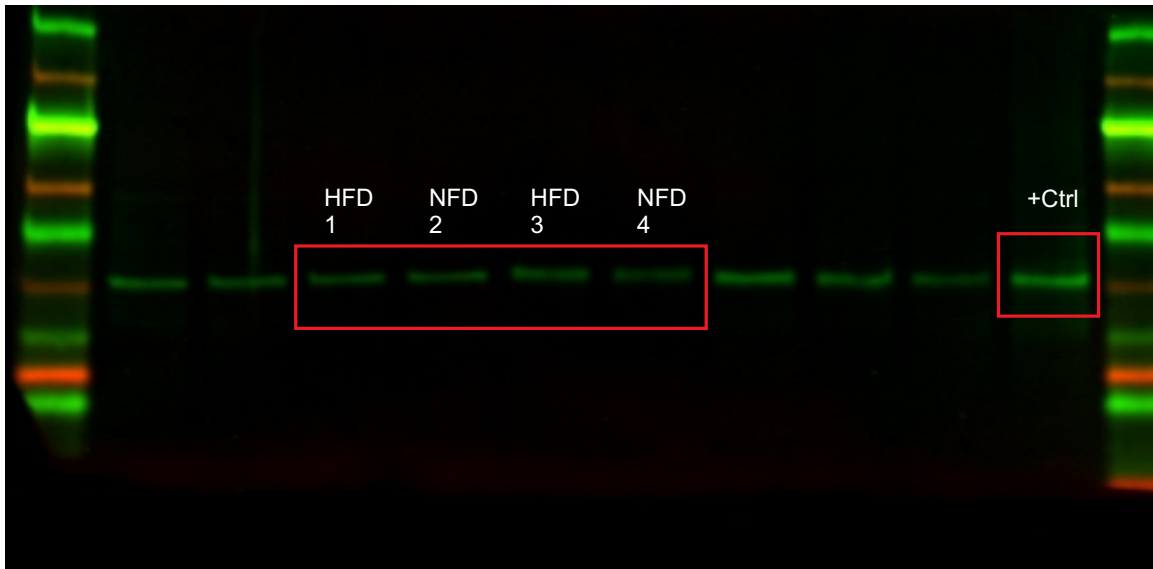

Supplement: Supplementary file 1 — Supplementary Information 1. [file 41598_2023_32492_MOESM1_ESM.pdf]

**Floxed- $\delta$**

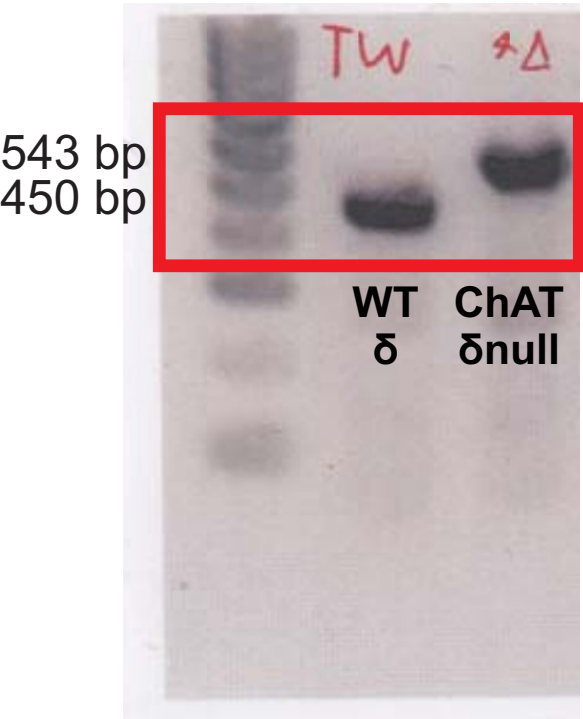

**ChAt-cre**

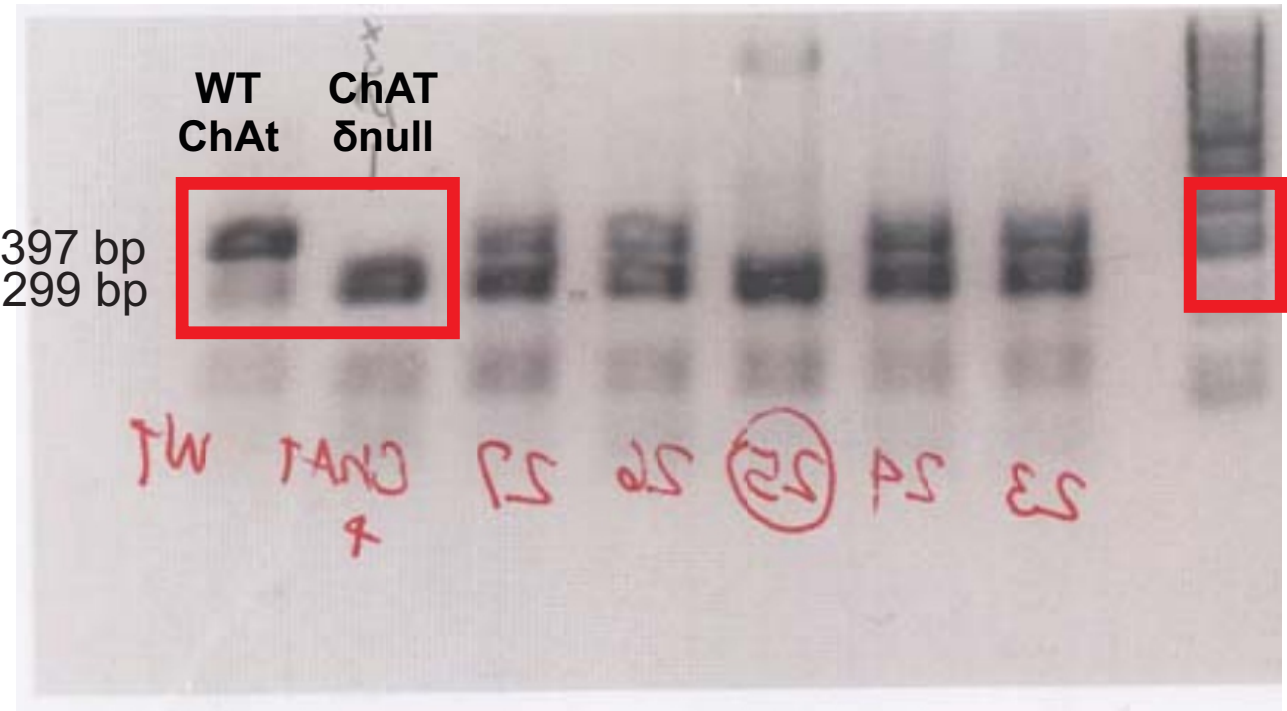

Supplement: Supplementary file 2 — Supplementary Information 2. [file 41598_2023_32492_MOESM2_ESM.pdf]
