# Supplementary material for: Early central cardiovagal dysfunction after high fat diet in a murine model
Source: Sci Rep. 2023 Apr 21;13:6550. doi: 10.1038/s41598-023-32492-w (PMC10121716; doi:10.1038/s41598-023-32492-w)
Supplement: Supplementary file 3 — Supplementary Information 3. [file 41598_2023_32492_MOESM3_ESM.docx]

**Supplemental Figure 1:** Original western blot/gel. Original image of GABA_A_(δ)R and α-tubulin loading control. Red box indicates the representative images used in Figure 3F. CB denotes positive control samples from the cerebellum where GABA_A_(δ)R expression is high.

**Supplemental Figure 2:** Original PCR gel. Original image of Foxed-δ and ChAt-cre PCR gels from Figure 4A.
